# Sputtering Deposited CuCrO_2_ and CuCrO_2_-ZnSnN_2_ Heterojunctions

**DOI:** 10.3390/nano16070416

**Published:** 2026-03-30

**Authors:** Xing-Min Cai, Yu-Feng Mei, Jian-Lin Liang, Wan-Fang Xiong, Fan Ye

**Affiliations:** 1 Key Laboratory of Optoelectronic Devices and Systems of Ministry of Education and Guangdong Province, Shenzhen Key Laboratory of Advanced Thin Films and Applications, College of Physics and Optoelectronic Engineering, Shenzhen University, Shenzhen 518060, China; 2 State Key Laboratory of Radio Frequency Heterogeneous Integration, Shenzhen University, Shenzhen 518060, China

**Keywords:** CuCrO_2_, ZnSnN_2_, heterojunction

## Abstract

There has been no experimental work on CuCrO_2_-ZnSnN_2_ heterojunctions (HJs), though theoretical work shows that their photoelectric conversion efficiency is around 20%. Here, CuCrO_2_ thin films and p CuCrO_2_-n ZnSnN_2_ HJs are prepared by varying the sputtering power of the Cu-Cr alloy target while the other parameters are held constant. The as-deposited Cu_x_Cr_y_O_z_ thin films are amorphous, with CuCrO_2_ as the major phase. The CuCrO_2_ thin films are p-type conductive, with an optical band gap of about 3.64–3.84 eV. The ZnSnN_2_ thin films are wurtzite and n-type conductive. The dark current density J versus voltage V curve measurements show that all the HJs showed rectification, while only the samples deposited at 40 and 50 W had a photo-induced current. Further analysis shows the HJs deposited at 40 W have the lowest shunt conductance, saturation current density, and trap density, implying an effect of fabrication conditions on the properties of HJs.

## 1. Introduction

CuCrO_2_ is a p-type semiconductor with a delafossite structure and a direct band gap of 2.95–3.30 eV [1,2,3,4,5,6,7,8,9,10,11]. Its application areas [1,2,3,4,5,6,7,8,9,10,11] include transparent electronic devices, photoelectrodes, catalysis and photocatalysis, gas and temperature sensing, magnetic and electrical energy storage, oxygen storage, water reduction, thermoelectricity, superconductivity, dye-sensitized or thin film solar cells, etc. Research has been conducted on CuCrO_2_. In 2014, a research team led by K.C. Sanal [2] deposited transparent p-type amorphous ${Cu}_{1-x}{Cr}_{x}O_{2-\delta }$ thin films on glass substrates using radio frequency (RF) magnetron co-sputtering at room temperature. In 2018, H. Zhang et al. [3] developed inverted perovskite solar cells with CuCrO_2_ nanocrystals as the hole transport layer (HTL), which were obtained through low-temperature solution processing, and achieved a high steady-state photoelectric conversion efficiency (PCE) of 19.0%. In 2022, the team of Fusheng Li [4] reported a simple, low-cost, and highly reproducible method for depositing a copper-poor CuCrO_2_ film through spray pyrolysis deposition. In 2023, a research team led by Sreeram Sundaresh [5] investigated the effect of annealing temperature (600–900 °C in N_2_ atmosphere) on the electrical, optical, structural, and morphological properties of CuCrO_2_ thin films. Their study showed the presence of the Cu^+^ oxidation state in pure CuCrO_2_ films. In 2024, a research team led by Selma Rabhi [6] reported the use of computational modeling to demonstrate that Mg-doped CuCrO_2_ thin films serve as an efficient HTL in perovskite solar cells (PSCs), and the optimized devices exhibited a PCE of approximately 22% under both front and rear illumination, highlighting their potential for high-performance applications. Recently, CuCrO_2_ heterojunctions with high breakdown voltage and low leakage current have also been demonstrated [10].

Despite the fact that there has been research about CuCrO_2_, including some theoretical work [12,13,14], there are still limited experimental studies on pn heterojunctions integrating CuCrO_2_ as the p-type layer and other semiconductors as the n-type layer. ZnSnN_2_ is a new n-type semiconductor and could potentially be used as a solar cell absorption layer due to its advantages, including its direct band gap, its high absorption coefficient, the abundance of its elements on Earth, and its lack of toxicity [15,16,17,18,19,20,21,22]. In 2018, E. Arca et al. theoretically showed that the photoelectric conversion efficiency of CuCrO_2_-ZnSnN_2_ heterojunction solar cells is 23.5%, based on the wx-AMPS simulation [12]. In their work, CuCrO_2_ was doped with Mg [12]. In 2020, A. Laidouci et al. also showed that, without Mg doping, the photoelectric conversion efficiency of the heterojunction is 22%, based on a simulation with SCAPS-1D [13]. Recently, it has also been shown that the photoelectric conversion efficiency is 18% [14]. However, there has been no experimental work about CuCrO_2_-ZnSnN_2_ heterojunctions. In this paper, the properties of CuCrO_2_ thin films deposited at different sputtering powers are first studied. Subsequently, CuCrO_2_-ZnSnN_2_ heterojunctions are prepared and the properties are revealed.

## 2. Experiments

### 2.1. Material and Device Fabrication

CuCrO_2_ thin films were deposited with direct current (DC) sputtering at room temperature. To study the properties of CuCrO_2_, K9 glass was used as the substrate. The substrates were cleaned in acetone, ethanol, and deionized water, and the washing time was 15 min for each liquid. The target was a copper–chromium alloy (99.999% purity; Tianqi Advanced Materials Co., Ltd, Beijing, China) with the Cu/Cr atomic ratio being 1. The DC sputtering power was 30–50 W. The sputtering chamber was evacuated to 5.0 × 10^−4^ Pa before film deposition. Ar (99.99%) and O_2_ (99.999%) were then introduced to the chamber, and the chamber pressure was maintained at 0.7 Pa during sputtering. The flow rate of Ar was 3 standard cubic centimeters per minute (sccm), and that of O_2_ was 7 sccm. Before film deposition, the target was sputtered for 5 min. The deposition time for CuCrO_2_ was 2 h. During deposition, the substrates were not intentionally heated and rotated with the substrate holder at 0.6π rad/s. The DC sputtering power was 30–50 W, and 5 batches of CuCrO_2_ thin films were prepared.

Radio frequency (RF) magnetron sputtering was used to prepare ZnSnN_2_, and the power was 30 W. The target for depositing ZnSnN_2_ was a high-purity zinc–tin (99.999%) alloy plate with a Zn/Sn atomic ratio of 4:1. The substrate temperature was maintained at 100 °C during deposition, and the rotational speed of the substrate holder was the same as that for depositing CuCrO_2_. The flow rates for Ar (99.99%) and N_2_ (99.999%) were 8 and 5 sccm, respectively. The work pressure was 5 Pa. The target was sputtered for 5 min to clean the surface before depositing ZnSnN_2_. The deposition time for ZnSnN_2_ was 3 h.

**Figure 1:**
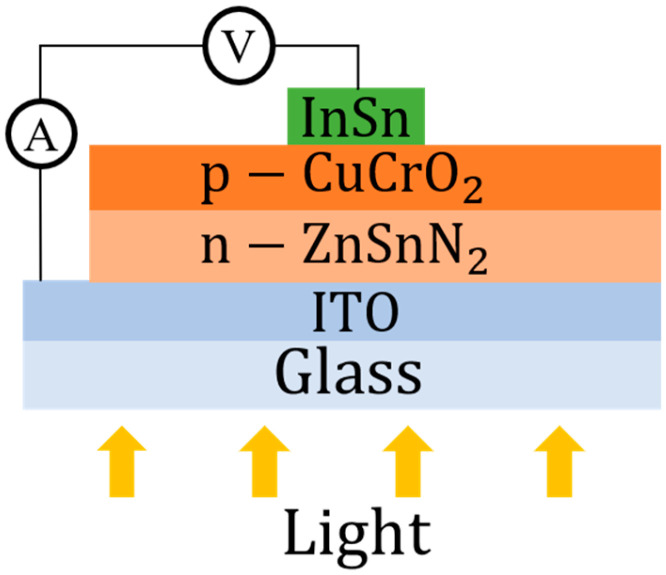
The cross-sectional diagram of the CuCrO_2_-ZnSnN_2_ heterojunction (glass\ITO\ZnSnN_2_\CuCrO_2_\InSn).

A schematic diagram of the CuCrO_2_-ZnSnN_2_ heterojunctions is shown in Figure 1. Indium tin oxide (ITO) coated glass of 20 × 20 mm^2^ was used as the substrate. The parameters for depositing ZnSnN_2_ and CuCrO_2_ were exactly the same as those mentioned in the previous two paragraphs. Before depositing ZnSnN_2_ on ITO, a shadow mask of the same size as the substrate was placed on the substrate. The shadow mask had nine 3 × 3 mm^2^ square apertures through which ZnSnN_2_ was deposited. After depositing ZnSnN_2_, CuCrO_2_ was then deposited on the ZnSnN_2_ (and the effective area of the heterojunctions was 3 × 3 mm^2^). Finally, a melted alloy of In and Sn (the In/Sn atomic ratio is 1.1) was pasted on the CuCrO_2_ layer to act as electrodes. By varying the sputtering power of CuCrO_2_, 5 batches of heterojunctions with CuCrO_2_ deposited at 30, 35, 40, 45, and 50 W were prepared.

### 2.2. Material and Device Characterization

The thickness of the films was measured using a surface profiler (Veeco Dektak 3ST). The crystal structure of the films was characterized with X-ray diffraction (XRD, Rigaku Ultima IV, θ–2θ scan). The electrical properties of the films were evaluated through Hall effect measurements (HL 5500 PC system) using the Van der Pauw method, with molten In-Sn alloy electrodes applied at the four corners of square samples. The optical properties, including reflectance and transmittance, were measured using a UV/VIS/NIR spectrophotometer (PerkinElmer Lambda 900). The chemical states of elements in the CuCrO_2_ thin films were analyzed by X-ray photoelectron spectroscopy (XPS, Thermo Fisher ESCALAB Xi+, Al Kα radiation, 1486.7 eV), where the C 1s peak was calibrated to be at 284.80 eV. Prior to the XPS measurements, the samples were surface-cleaned by Ar^+^ ion sputtering for 25 s at a relative etch rate of 0.25 nm/s (referenced to Ta_2_O_5_). The dark and photovoltaic performance were assessed by measuring the current density J versus the voltage V (JV) under dark or illuminated conditions (AM 1.5G, 100 mW/cm^2^) using a Keithley 2400 source meter coupled with a solar simulator (Zolix SS150, Zolix, Beijing, China) which was calibrated with a standard Si reference cell.

## 3. Results and Discussion

**Figure 2:**
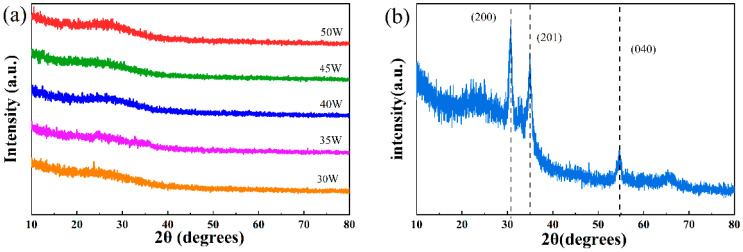
(**a**) The XRD patterns of CuCrO_2_ films deposited at room temperature; (**b**) the XRD pattern of ZnSnN_2_ thin film.

Figure 2a shows the XRD spectra of the CuCrO_2_ films deposited at room temperature. All the thin films deposited at different sputtering powers had no diffraction peaks, indicating an amorphous structure and in agreement with the literature [8]. This resulted from the fact that the substrates were not heated during deposition. It is reported that after annealing the Cu_x_Cr_y_O_z_ films, which were deposited at room temperature, at 550 °C and 575 °C, both CuO and CuCr_2_O_4_ could be detected, while after annealing the Cu_x_Cr_y_O_z_ films at 600 °C, CuCrO_2_ emerged as the dominant phase, with CuO as the minor phase [8]. In our work, post-deposition annealing was not conducted, since post-deposition annealing was not compatible with the heterojunction preparation. Figure 2b shows the XRD pattern of ZnSnN_2_. Weak diffraction peaks were observed, implying poor crystallization, and ZnSnN_2_ could be indexed as wurtzite with grain sizes at the nanometer level [17,18,19].

**Figure 3:**
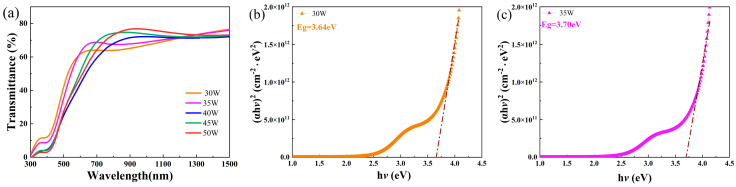

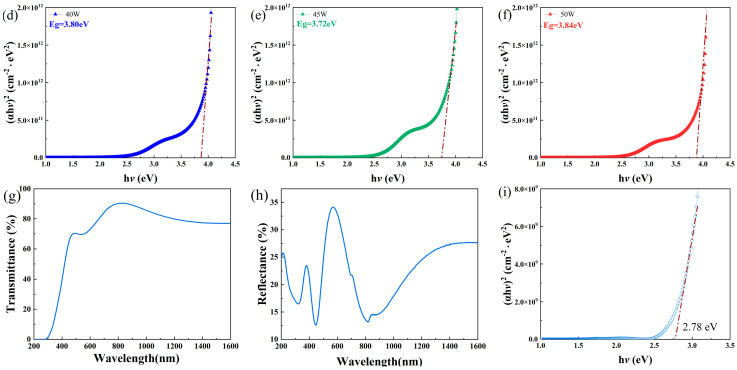
The transmittance and Tauc plots of the samples deposited at different sputtering powers. (**a**) Transmittance spectra; (**b**–**f**) the Tauc plots of the samples with CuCrO_2_ deposited at 30–50 W. The transmittance (**g**) and reflectance spectrum (**h**) of ZnSnN_2_; (**i**) the Tauc plot for ZnSnN_2_.

The thickness of CuCrO_2_ films deposited at 30, 35, 40, 45, and 50 W was 102.1, 129.5, 199.1, 163.5, and 196.3 nm, respectively. Hall effect measurements showed that CuCrO_2_ films were p-type-conductive, and the hole density was about 1.07 × 10^10^ cm^−3^. Cu vacancies, together with oxygen interstitials, were possibly the major acceptors according to theoretical work [23,24,25]. The transmittance and optical band gap of the CuCrO_2_ samples varied with the sputtering power, as shown in Figure 3a–f. The CuCrO_2_ films prepared under different sputtering powers had similar transmittance. The transmittance of the films at 750 nm was between 60% and 75%, which is consistent with the literature [7]. The absorption coefficient α can be calculated from the transmittance T_r_ and reflectance R_r_ data, since α = *d*^−1^ln[(100 − *R_r_*)/*T_r_*], where *d* is the film thickness. The relation between *α* and the optical band gap E_g_ for CuCrO_2_ is (*αh*ν)^2^ = *A_L_*(*h*ν − E_g_), where *A_L_* is a constant, *h* is the Plank constant, and ν is the photon frequency [26]. From the (*αh*ν)^2^ versus *h*ν curves or the Tauc plots in Figure 3b–f, the effective optical band gap E_g_ can be obtained by extrapolating the linear region to intercept the *h*ν axis [26]. The effective optical band gaps of the samples prepared under sputtering powers of 30, 35, 40, 45, and 50 W are 3.64, 3.70, 3.80, 3.72, and 3.84 eV, respectively. The band gap was larger than in other work (2.1–2.8 eV [2]; 3.61 eV [11]) due to the amorphous nature of the samples. The optical characterization of ZnSnN_2_ is shown in Figure 3g–i. The thickness of ZnSnN_2_ was 228 nm. Similarly, the band gap of ZnSnN_2_ was found to be 2.78 eV. The electron concentration of ZnSnN_2_ was measured to be 9.63 × 10^17^ cm^−3^.

**Figure 4:**
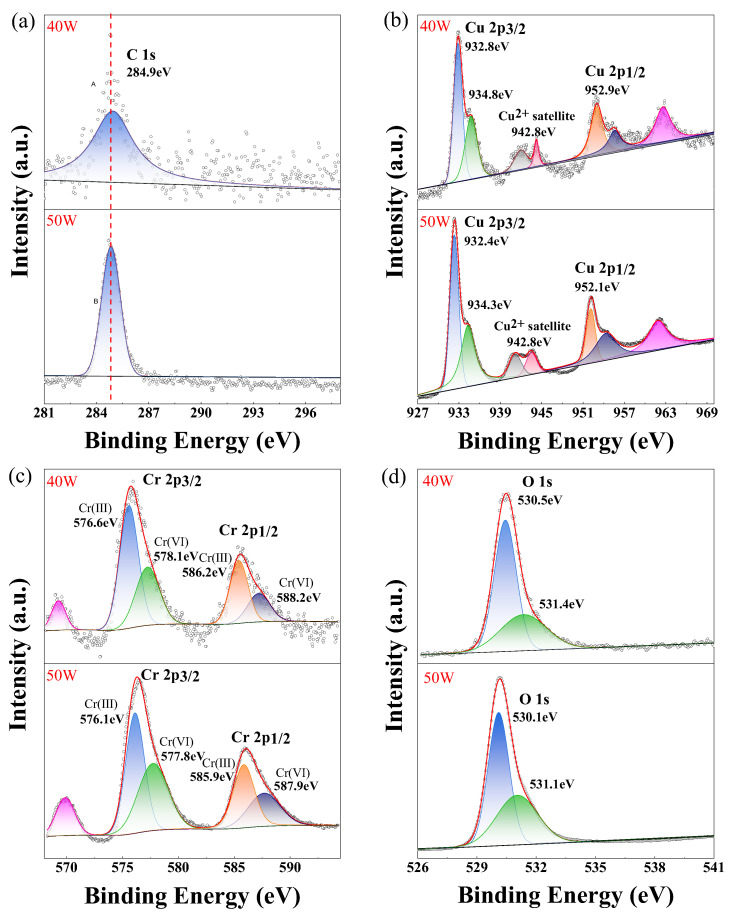
High-resolution XPS energy spectra of C, Cu, Cr, and O elements in the films deposited at 40 W and 50 W. (**a**) C; (**b**) Cu; (**c**) Cr; (**d**) O.

The chemical composition and electronic states of the glass/CuCrO_2_ thin films were characterized by X-ray photoelectron spectroscopy (XPS). The XPS survey spectra of CuCrO_2_ films deposited at room temperature with the sputtering powers of 40 W and 50 W show that the samples contain Cu, Cr, and O. The atomic percentages of Cu, Cr, and O were 21.48%, 20.50%, and 58.02%, respectively. Figure 4 displays the deconvoluted high-resolution XPS spectra of Cu 2p, Cr 2p, and O 1s for CuCrO_2_ films deposited at 40 W and 50 W. For both samples, the Cu 2p_1/2_ peak appears at approximately 952 eV, while the Cu 2p_3/2_ peak is located at around 932 eV [26,27,28]. The Cu 2p_3/2_ peak can be deconvoluted into two peaks: the stronger one, at 932 eV, corresponds to Cu^+^ cations, and the weaker one, at 934 eV, represents Cu^2+^ cations [26,27,28]. Previous studies [26,27,28] have demonstrated that copper chromium oxides primarily exist in two distinct phases: delafossite CuCrO_2_ and spinel CuCr_2_O_4_. In CuCrO_2_, copper exists in the +1 oxidation state (Cu^1+^), whereas in CuCr_2_O_4_, it is in the +2 oxidation state (Cu^2+^). These two copper oxidation states exhibit markedly different core-level spectral characteristics: Cu^2+^ spectra display intense shake-up satellite peaks, while such satellite features are absent in Cu^1+^ spectra, and these spectral differences serve as a reliable fingerprint for distinguishing these two copper oxidation states in mixed-phase systems [26,27,28]. As observed in Figure 4, the Cu 2p high-resolution XPS spectra of samples prepared at 40 W and 50 W exhibit similar satellite peaks at 943 eV. These results demonstrate the coexistence of both Cu^+^ and Cu^2+^ cations in the films, with the Cu^2+^ oxidation state likely originating from the CuCr_2_O_4_ phase. In the spectra in Figure 4c, the Cr-2p doublet can be observed. The Cr 2p_1/2_ and Cr 2p_3/2_ peaks are approximately at 586 eV and 576 eV, implying that the Cr in the films is at +3 [27,28]. Two peaks are observed in the deconvoluted O-1s high-resolution XPS spectra (Figure 4d). The major peak at about 530 eV corresponds to lattice oxygen, while the minor one at about 531 eV is due to adsorbed oxygen species [27].

**Figure 5:**
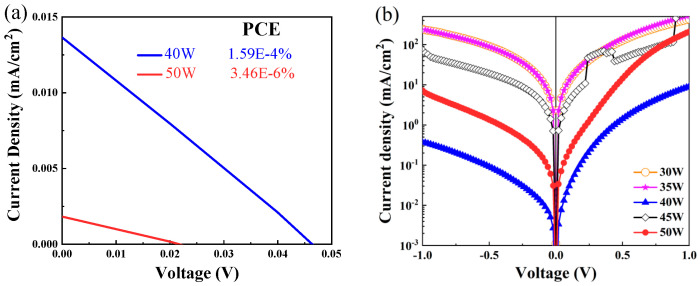
(**a**) The illuminated JV curves of CuCrO_2_-ZnSnN_2_ heterojunctions deposited at 40 W and 50 W; (**b**) the dark JV curves of all the CuCrO_2_-ZnSnN_2_ heterojunctions.

The illuminated and dark JV curves of CuCrO_2_-ZnSnN_2_ heterojunctions were measured and are presented in Figure 5. Only the heterojunctions deposited at 40 W and 50 W have photo-induced currents (Figure 5a). From the dark JV curves of all the samples (Figure 5b), rectification is observed. The heterojunction prepared at 40 W demonstrates a power conversion efficiency (PCE) of 1.88 × 10^−4^% with an open voltage (V_OC_) of 0.046 V, fill factor (FF) of 29.209%, and short-current density (J_SC_) of 0.014 mA/cm^2^, while the 50 W sample shows a PCE of 7.27 × 10^−6^% (V_OC_ = 0.022 V, FF = 16.529%, J_SC_ = 0.002 mA/cm^2^). The low PCE possibly mainly resulted from the band gap, which was not the optimal one. The thickness of ZnSnN_2_ was also insufficient. As shown in Figure 5b, all heterojunctions fabricated with sputtering powers ranging from 30 W to 50 W exhibited effective rectifying characteristics, confirming the formation of functional heterojunctions.

According to the single-diode model [29], the JV relation is

(1) $$J=J_{0}\exp\left[\frac{q}{AkT}\left(V-JR\right)\right]+GV-J_{L}.$$

**Figure 6:**
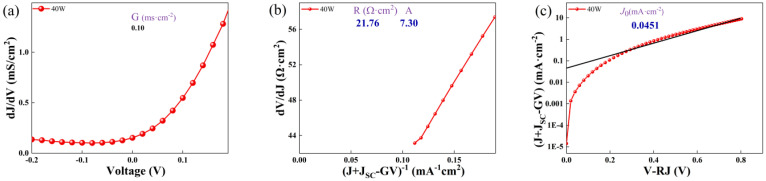
The analysis curves to extract the parameters of the CuCrO_2_-ZnSnN_2_ heterojunction deposited at 40 W. (**a**) dJ/dV–V curve; (**b**) dV/dJ–(J + J_SC_-GV)^−1^ curve; (**c**) ln(J + J_SC_-GV)–(V-RJ) curve.

Here, J_0_ represents the diode saturation current, q denotes the elementary charge, A stands for the ideality factor, k is the Boltzmann constant, T indicates temperature, R is the series resistance, G refers to the shunt conductance, and J_L_ is the light-induced current density. The method to extract these parameters is shown in Figure 6 (assuming J_L_ = 0 for dark JV curves). The shunt conductance (G) values can be extracted from the plateau regions of the dJ/dV versus V curves (Figure 6a). Subsequently, through the analysis of the dV/dJ versus (J + J_SC_-GV)^−1^ curves (Figure 6b), the values of the series resistance (R) and ideality factor (A) can be obtained, with the y-axis intercept providing the R value and the slope of AkT/q yielding the A value. Furthermore, the reverse saturation current (J_0_) was extracted from the ln(J + J_SC_-GV) versus V-RJ curves (Figure 6c). The obtained parameters for the four heterojunctions are summarized in Table 1.

As shown in Table 1, the samples deposited at 40 and 50 W have lower G and J_0_ compared with those deposited at 30 and 35 W, indicating lower defect densities both within the CuCrO_2_-ZnSnN_2_ layer and at its front/back interfaces, and suggesting improved carrier transport and reduced current leakage pathways. Smaller series resistance, R, corresponds to lower back-contact resistance and reduced back-contact potential barriers in solar cell devices. The samples deposited at 40 W have larger R values at 40 W, and this accounts for their significant V_OC_ losses and low fill factors (FF (%)) [19,30]. The ideality factors (A) exceeding 2 for the samples deposited at 40 and 50 W possibly indicate significant recombination and interface defects at the CuCrO_2_-ZnSnN_2_ interface. Of the four parameters, only the reverse saturation current (J_0_) differs by orders of magnitude. This substantial variation in J_0_ dominates the device performance, explaining why the samples deposited under 40 and 50 W conditions had photo-induced currents, as well as why the photovoltaic conversion efficiency of the heterojunction deposited at 40 W was higher than that at 50 W.

**Figure 7:**
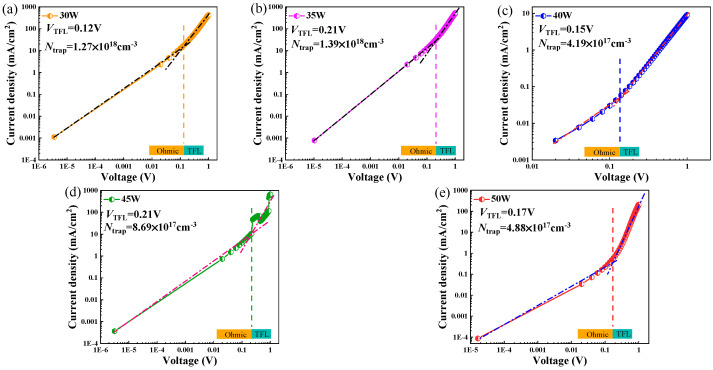
The forward-biased logarithmic JV curves of CuCrO_2_-ZnSnN_2_ heterojunctions fabricated under different sputtering powers. (**a**) 30 W; (**b**) 35 W; (**c**) 40 W; (**d**) 45 W; (**e**) 50 W.

From the forward-bias logarithmic JV curves of these samples (Figure 7), two regions can be clearly identified. In the low-voltage region (below about 0.14 V), the slope of the curve is close to 1, which clearly indicates that current is ohmic and controlled by the pn junction. As the voltage increases, the slope of the curve increases over 2, indicating the current is space-charge-limited (SCL) or in the mode of the trap filling limit (TFL) effect [19,31]. In this mode, the trap density N_trap_ can be estimated according to the following equation:

(2) $$N_{trap}=\frac{2\varepsilon_{0}\varepsilon_{r}V_{TFL}}{qL^{2}}.$$

Here, L is the thickness of the CuCrO_2_ film, ε_0_ is the vacuum dielectric constant, ε_r_ (9.5 [32]) is the relative dielectric constant, and V_TFL_ is the critical voltage for the presence of TFL. The value of V_TFL_ can be read in Figure 7, and the trap densities (N_trap_) of the heterojunctions deposited at 30 to 50 W were calculated to be 1.27 × 10^18^, 1.39 × 10^18^, 4.19 × 10^17^, 8.69 × 10^17^, and 4.88 × 10^17^ cm^−3^, respectively. Since the effect of the ZnSnN_2_ layer was ignored during the calculation, the calculated trap density can only be used as an order-of-magnitude estimation. The samples deposited at 40 W and 50 W had smaller trap densities.

**Figure 8:**
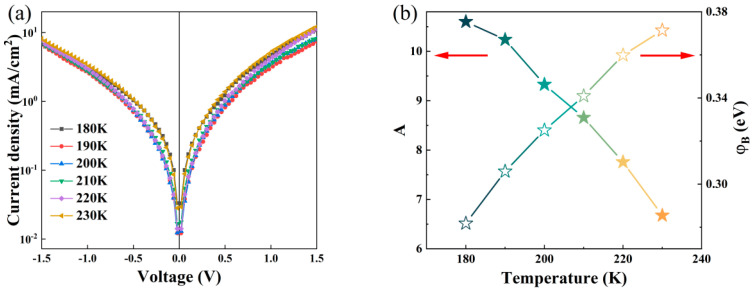
(**a**) Dark temperature-dependent JV curves of the CuCrO_2_/ZnSnN_2_ heterojunction deposited at 40 W. (**b**) The ideality factor A and the barrier height $\varphi_{B}$.

Dark JV measurements under varying temperatures were performed on the CuCrO_2_-ZnSnN_2_ heterojunction with CuCrO_2_ deposited at 40 W (Figure 8a). According to the thermionic emission (TE) model [33], the JV relationship is

(3) $$J=J_{0}\exp\left(\frac{qV}{AkT}\right)[1-\exp\left(\frac{-qV}{kT})\right],$$

where J_0_ represents the saturation current density, and other parameters are the same as previously defined. The saturation current density J_0_ can be expressed as

(4) $$J_{0}=A^{**}T^{2}\exp\left(\frac{-q\varphi_{B}}{kT}\right).$$

Here, φ_B_ is the barrier height, while A** represents the effective Richardson constant (the Richardson constant A** is given by $4\pi qm^{*}k^{2}h^{-3}$; m* is the effective mass of charge carriers and m* = 3.8m_0_, where m_0_ is the electron rest mass [34]).

From Equation (3), Equation (5) can be obtained as follows:

(5) $$ln\{J/[1-exp(-\frac{qV}{kT})]\}=\frac{qV}{AkT}+lnJ_{0}.$$

The temperature-dependent JV curves of the sample can be processed according to Equation (5). From the slope of $ln\{J/[1-exp(-\frac{qV}{kT})]\}$ versus V curves, the ideality factor A can be obtained, while the saturation current density J_0_ can be obtained from the intercept on the vertical axis. The barrier height $\varphi_{B}$ can then be calculated according to Equation (4). The obtained ideality factor A and barrier height $\varphi_{B}$ are in Figure 8b.

The barrier height increases while the ideality factor decreases with an increase in temperature, implying barrier height inhomogeneity. The reduction in the ideality factor indicates significant suppression of interface recombination effects at elevated temperatures. This decreasing trend primarily originates from the defect state density distribution characteristics at the CuCrO_2_-ZnSnN_2_ heterojunction interface. At low temperatures, unsaturated dangling bonds at the interface form recombination centers, resulting in a larger ideality factor A. With increasing temperature, the thermal activation of carrier filling of interface states leads to gradual passivation of recombination channels, thereby reducing the A value [35]. The anomalous increase in the barrier height ($\varphi_{B}$) primarily originates from the spatial inhomogeneity of barrier potential, coupled with dielectric confinement effects and transitions in carrier transport mechanisms [36]. As temperature increases, the enhanced interfacial polarization effect partially offsets the image-force-induced barrier lowering, resulting in a net increase in the apparent barrier height. At lower temperatures, the conduction is dominated by variable-range hopping (VRH) through low-barrier defect clusters, while rising temperatures broaden the carrier energy distribution, enabling thermally activated carriers to overcome higher potential barriers and extend current pathways to higher-barrier regions. This transition in transport mechanisms effectively modifies the statistical weighting of the conduction channels, leading to the observed increase in $\varphi_{B}$, which fundamentally reflects the intrinsic inhomogeneity of the barrier height distribution at the heterointerface [36].

**Figure 9:**
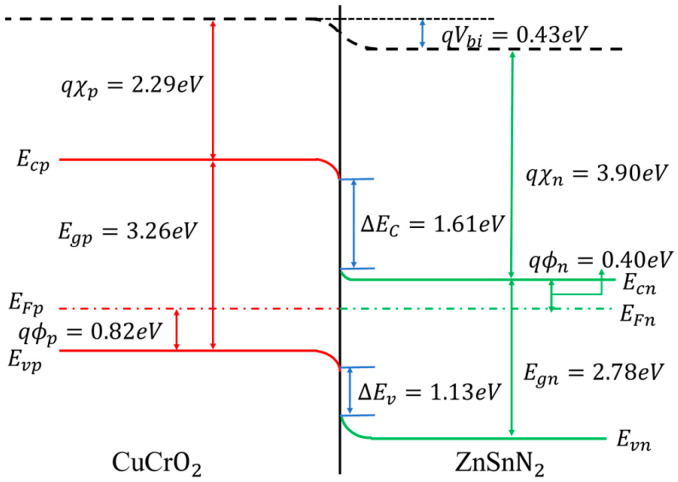
The energy band diagram of the CuCrO_2_-ZnSnN_2_ heterojunction.

Figure 9 shows the energy band diagram of CuCrO_2_-ZnSnN_2_. The band gap of ZnSnN_2_ is 2.78 eV. The electron concentration of ZnSnN_2_ is 9.63 × 10^17^ cm^−3^, and the energy difference between the conduction band minimum and Fermi level (E_C_ − E_F_) is calculated to be 0.40 eV [18,37]. For the p-type CuCrO_2_ thin films, the effective hole mass is m_p_* = 3.8m_0_ [38]. At room temperature, the effective density of states in the valence band (N_V_) is 4.69 × 10^19^ cm^−3^. The energy difference between the Fermi level and valence band maximum (E_F_ − E_V_) is 0.82 eV with the hole concentration p_0_ = 1.07 × 10^10^ cm^−3^. The band gap of CuCrO_2_ thin films used here is 3.26 eV (which is near that used in the simulation [14]). The electron affinity of ZnSnN_2_ thin films is 3.90 eV [19,21,38], and that of CuCrO_2_ thin films is 2.29 eV [32]. With these parameters, the built-in potential is 0.43 eV. The conduction band offset is 1.61 eV and the valence band offset is 1.13 eV. The large band offset will result in strong interface recombination, which could possibly be reduced with interfacial passivation layers such as AlN. The band diagram is not optimal to obtain high PCE. The band gap of ZnSnN_2_ here is much larger than the optimal band gap (1.5 eV) and this is the major reason for why the efficiency is much lower than the calculated results [12,13,14]. Another reason is that both CuCrO_2_ and ZnSnN_2_ lack crystallinity. This results in lower mobility, larger defect density, and, finally, poorer photovoltaic performance. More work is needed in optimizing the properties of both CuCrO_2_ and ZnSnN_2_ in order to improve the PCE of CuCrO_2_-ZnSnN_2_ heterojunctions.

## 4. Conclusions

In summary, firstly, the preparation of CuCrO_2_ was studied. CuCrO_2_ thin films were prepared with DC magnetron sputtering at different sputtering powers. XRD showed that the as-deposited films were amorphous due to room temperature deposition. The optical band gap was in the range of 3.64–3.84 eV. XPS measurement showed that CuCrO_2_ was the dominant phase. CuCrO_2_ films were p-type conductive. Secondly, CuCrO_2_-ZnSnN_2_ heterojunctions with CuCrO_2_ deposited at different sputtering powers were prepared, and their properties were characterized. The dark JV curves of all the heterojunctions showed rectification, while the samples deposited at 40 and 50 W had photo-induced currents due to their relatively lower diode saturation current density, lower shunt conductance, and smaller trap density. The barrier heights of the heterojunctions were inhomogeneous, with the barrier height increasing and ideality factor decreasing as the measurement temperature increased. Since the device performance was poor, the extracted parameters, including diode parameters, trap densities, and barrier heights, might represent a relative trend rather than the absolute device quality. The energy band diagram of CuCrO_2_-ZnSnN_2_ heterojunctions was not optimal to obtain higher PCE, and further work is needed in optimizing the properties of CuCrO_2_ and ZnSnN_2_ in order to improve their PCE.

## Figures and Tables

**Table 1:** The shunt conductance (G), series resistance (R), ideality factor (A), and reverse saturation current density (J_0_) of the CuCrO_2_-ZnSnN_2_ heterojunctions.

Sputtering Power (W)	G (mS·cm^−2^)	$J_{0}$ (mA·cm^−2^)	R (Ω·cm^2^)	A
30	114	6.35 × 10^−1^	1.64	1.11
35	114	6.90 × 10^−1^	1.30	1.14
40	0.10	4.51 × 10^−2^	21.76	7.30
50	1.49	4.82 × 10^−2^	1.37	3.52

## Data Availability

The original contributions presented in this study are included in the article. Further inquiries can be directed to the corresponding author.
